# A 19 day earth tide measurement with a MEMS gravimeter

**DOI:** 10.1038/s41598-022-16881-1

**Published:** 2022-07-29

**Authors:** Abhinav Prasad, Richard P. Middlemiss, Andreas Noack, Kristian Anastasiou, Steven G. Bramsiepe, Karl Toland, Phoebe R. Utting, Douglas J. Paul, Giles D. Hammond

**Affiliations:** 1 School of Physics and Astronomy, University of Glasgow, Kelvin Building, University Avenue, Glasgow, G12 8QQ UK; 2 James Watt School of Engineering, University of Glasgow, Rankine Building, Oakfield Avenue, Glasgow, G12 8LT UK

**Keywords:** Natural hazards, Applied physics, Electronics, photonics and device physics, Techniques and instrumentation, Techniques and instrumentation, Electrical and electronic engineering

## Abstract

The measurement of tiny variations in local gravity enables the observation of subterranean features. Gravimeters have historically been extremely expensive instruments, but usable gravity measurements have recently been conducted using MEMS (microelectromechanical systems) sensors. Such sensors are cheap to produce, since they rely on the same fabrication techniques used to produce mobile phone accelerometers. A significant challenge in the development of MEMS gravimeters is maintaining stability over long time periods, which is essential for long term monitoring applications. A standard way to demonstrate gravimeter stability and sensitivity is to measure the periodic elastic distortion of the Earth due to tidal forces—the Earth tides. Here, a 19 day measurement of the Earth tides, with a correlation coefficient to the theoretical signal of 0.975, has been presented. This result demonstrates that this MEMS gravimeter is capable of conducting long-term time-lapse gravimetry, a functionality essential for applications such as volcanology.

## Introduction

Gravimetry has been used extensively over the past century in several fields. Due to the high cost of commercial instruments (around £70k for a portable device) the oil and gas industry has been the most prolific user of gravimetry, where it is often used as a pre-drilling survey method to investigate subterranean geological features^1,2^. Gravimetry has also been used, however, for sinkhole analysis^3^, finding tunnels and cavities for the defence sector^4,5^, CO$_2$ sequestration^6^, geothermal reservoir monitoring^7^, archaeology^8^, hydrology^9,10^, and volcanology^11–15^.

The last item in this list is perhaps the field in which gravimetry could offer the most societal benefit, but for which its use has been severely limited by the high capital cost of the equipment. Gravimetry is the only means by which mass variations within volcanoes can be measured. Gravimetry therefore offers a great advantage to hazard forecasting because mass changes often precede eruptive processes^12,16,17^. Freire et al.^18^ state that ‘more than 8% of the world’s 2015 population lived within 100 km of a volcano with at least one significant eruption’. The significance to humanity of improving eruption forecasting cannot therefore be overstated. Gravimetry has been used to a limited extent in this setting, but spatial resolution has been limited by the number of instruments that can be affordably bought. Whilst single sensors can only provide a point measurement, if the price of gravimeters could be significantly reduced then multi-pixel gravity imaging would become possible. An analogy could be drawn between this and the example of a digital camera; with a single stationary pixel you cannot capture a picture, you only have a light sensor, but this soon changes as you gain pixels. In 2019 Carbone et al.^19^ described a measurement using only three sensors on Mt. Etna as an ‘network’; such is the sparsity of gravimeters on what is one of the most studied volcanoes in the world. With this application in mind, the authors have joined a consortium (NEWTON-g) to develop a gravimeter network on Mt. Etna comprising tens of MEMS gravimeter ‘pixels’^20^, and a MuQuans Absolute Quantum Gravimeter^21^.

Due to the inverse square law of gravity, a single measurement does not have a single unique solution; it is impossible to tell whether a signal has a source that is big and far away, or small and close. Inversion techniques therefore need to be applied to understand the data. Arrays such as the planned network on Mt. Etna present a great opportunity to reduce the problem of inversion, because by using two or more sensors one benefits from triangulation in reducing the number of possible solutions. The more sensors one introduces, the more information one receives. In essence, the data from an array of sensors is greater than the sum of its parts; and multi-pixel gravity imaging has the potential to change the way that mass changes within volcanoes are monitored.

In order to expand the use of gravimetry and to enable novel multi-pixel/array-based applications, it is imperative to develop a new gravimeter technology that is both affordable as well at par with the state-of-the-art commercial gravimeters in terms of sensitivity and stability. Over the past 8 years the authors have developed a miniaturised gravity sensor that is based on the fabrication processes used to make MEMS (microelectromechanical system) accelerometers found in smart phones. MEMS can be reproduced in large volumes, with low cost, and with remarkable fabrication tolerances. Historically, MEMS devices were dismissed for use in gravimetry, since most application required long-term measurements of days to weeks, and this long-term stability could not be maintained for existing MEMS devices.

In 2016, the authors demonstrated, for the first time, the measurement of Earth Tides using a MEMS device^22^. Given its remarkable stability, the device was the first true MEMS gravimeter. Since then, the field has become more active and several other teams have published results on different designs of MEMS gravimeters^23–25^. In this paper, the authors are proposing a new generation of Glasgow’s relative MEMS gravimeter that demonstrates a significant improvement in performance with a highly accurate Earth Tide measurement over 19 days (more than three times longer than any other published data from any MEMS device). This data aligns with a theoretical signal with a correlation coefficient of 0.975. The new device uses a capacitive readout scheme that has a higher displacement sensitivity compared to the previously used optical shadow sensing scheme. This allowed an increase of resonant frequency of the device to 7.35 Hz, making the device more robust compared to its previous 2.3 Hz counterpart. With the new MEMS sensor, it has been estimated to have a bias instability of 8.18 $\upmu$Gal for an averaging period of 417 s, and a bias instability of <1 $\upmu$Gal over an averaging period of 250 s when considering the gravimeter’s electronic noise alone. This low bias instability has been achieved by undertaking the measurements in a lab situated at the West End of Glasgow City and without subtracting seismic noise^24,25^. A linear drift rate of <268 $\upmu$Gal/day of the new device is also reported, which is one of the best reported in the MEMS gravimeter class (see the performance comparison Table S1 in the supplementary information).

## Design of the MEMS gravimeter

The MEMS gravimeter has two distinct components—a monolithically etched silicon sensor with a high acceleration sensitivity; and an integrated capacitive readout scheme to measure the sensor’s output. Here, the design aspects of the core MEMS sensor will be discussed briefly, followed by an explanation of the implemented readout scheme.

**Figure 1:**
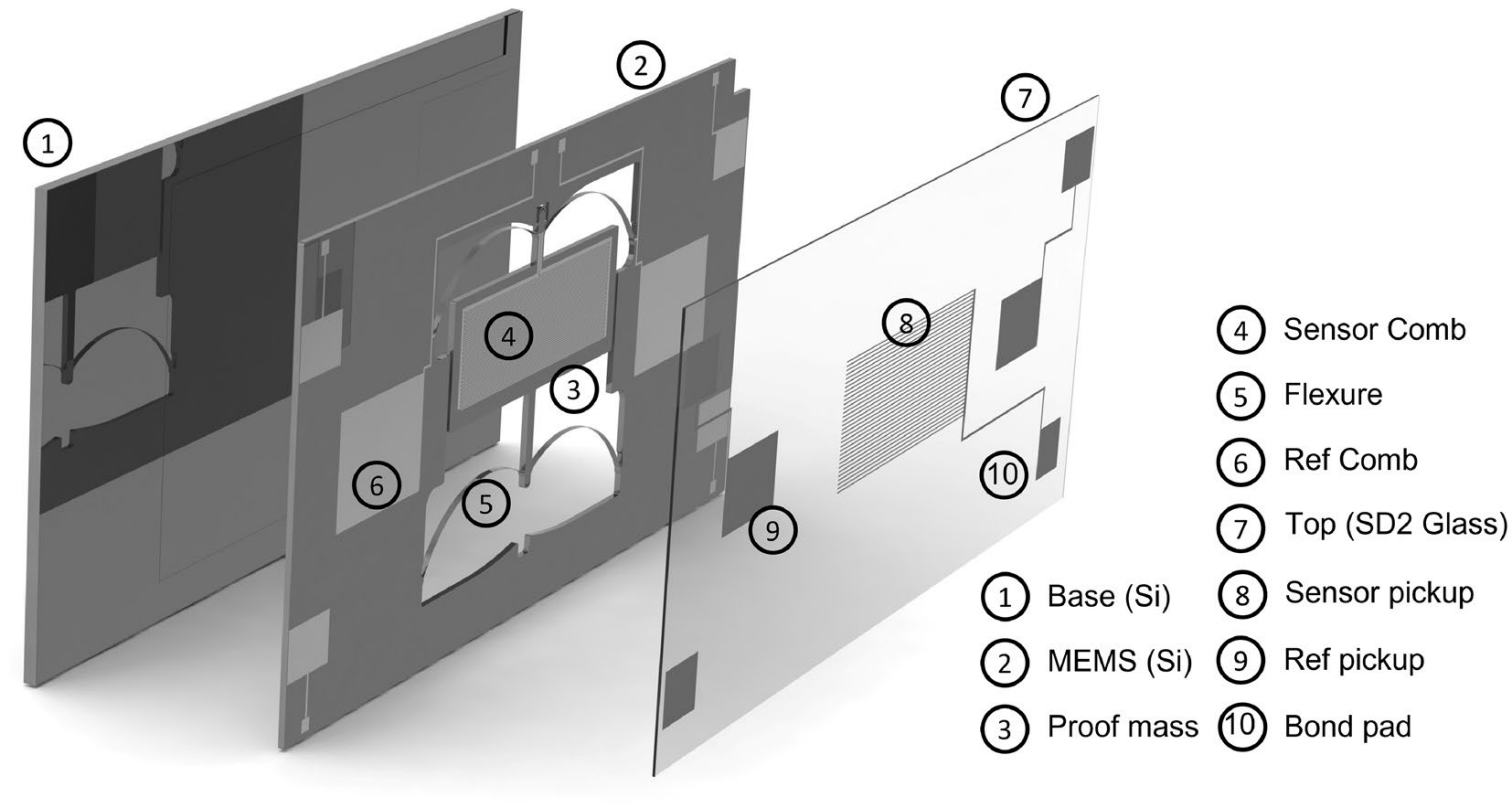
The MEMS device. An exploded view of the MEMS gravimeter assembly. The middle MEMS layer is mounted upon a base plate. The silicon base plate simply serves as a mechanical support for the MEMS layer. The MEMS layer has metal capacitive electrodes (combs) patterned on its surface. Corresponding pickup electrodes are patterned on an upper glass layer (made from SD2 silica), which is used to read out the signal as the MEMS proof-mass moves. A couple of reference capacitors were fabricated on the frame of the MEMS layer to monitor the gap/lateral movement between the assembly layers of the device.

The design of the sensor is a based upon the device first presented by the group in 2016^22,26,27^. Significant changes have been made since this time, however, in order to miniaturise the sensor and improve both its sensitivity and stability. The sensor is a relative gravimeter: it is able to measure changes in gravity by monitoring the relative displacement of a proof-mass on a spring. For an oscillating system such as this, the input acceleration, *a*, and the corresponding proof-mass displacement, *z*, are directly proportional to each other through the relationship $a = 4\pi ^2f^2 z$, where *f* is the fundamental resonant frequency, and the bandwidth of the sensor. Here, the mass and the supporting springs are etched monolithically from a single piece of silicon using standard photolithography and etching techniques^28^ (see Fig. 1 for a CAD of the sensor and Fig. 6b for a photo of the MEMS layer). The four symmetric springs utilise a geometric anti-spring design^29–33^. The details of how this design can be tuned for the purposes of gravimeter fabrication is detailed in the paper by Middlemiss et. al.^34^. This design enables a decreased spring stiffness in the vertical direction whilst limiting the motion in the horizontal and out-of-plane axes. Decreasing the stiffness—and hence the oscillation frequency—in the operation axis is important because it means that a proof-mass will move more for a given change in gravity. This is particularly useful in cases where the sensitivity of the system is limited by the displacement readout. Softening the spring, however, comes at a cost because the robustness of the sensor decreases as the resonant frequency drops. With the intention of developing a much more robust sensor, the spring design was optimised to obtain a working resonant frequency of 7.35 Hz (compared to 2.2 Hz in earlier publications^22^). Given that there was also a desire to improve on the sensitivity reported in the earlier work, it was necessary to improve the displacement sensitivity measurement of the proof-mass.

The measurement of the MEMS displacement is, of course, another fundamental function of the device. In Middlemiss et al.^22^, an optical shadow sensor was used to measure the displacement of the mass^35^. An LED was used to cast light on a photodiode, and the MEMS was placed in the light beam. As the mass moved, the shadow cast on the photodiode altered the photocurrent, which was used as a proxy for displacement. This methodology was limiting for several reasons. A displacement sensitivity of less that 1 nm was difficult to achieve, ultimately limiting the acceleration sensitivity of the overall sensor. The components needed to be mounted on a bespoke machined block of fused silica to reduce temperature sensitivity. The disadvantage of this block was twofold: it was expensive to machine, and it was far too big to fit inside a standard MEMS package. Whilst this device configuration was used to conduct field tests^36–38^, further miniaturisation was required.

To facilitate the device miniaturisation, a capacitive displacement method was adopted to readout the motion of the proof-mass. A set of metal comb electrodes were patterned on the top surface of the proof-mass while a complementary set of electrodes were patterned on a fixed glass layer that was separated from the proof-mass layer by a gap of 30–40 $\upmu$m using SU8 spacers. The combs on the two layers formed overlapping capacitors. While the proof-mass comb was electrically driven by a 40 kHz differential pair of sine waves, the signal was picked up by the set of fixed combs on the glass layer. Any displacement of the proof-mass modulates the overlapping capacitance and, thus, the output current flowing through the capacitors. Therefore, by monitoring the changing overlapping capacitance, the position of the mass could be measured. The current obtained at the output of the overlapping capacitors was fed to an operational amplifier that was configured in the conventional transimpedance amplification (TIA) topology. The transimpedance topology, despite its simplicity, is very effective in reading current from high impedance sources because its signal gain is insensitive to capacitive parasitics at the pick-up stage. The noise gain of the TIA stage, however, is a function of the input parasitics and needs more attention during circuit design when the sensitivity of the system is limited by the electronics noise floor. The voltage signal obtained after the amplification stage was then fed to a bench-top lock-in amplifier. The signal was demodulated and low-pass filtered, and the signal amplitude that carried the displacement information was acquired and logged onto a PC through a Labview routine at a sampling frequency of roughly 0.2 Hz. Using the modulation-demodulation lock-in approach to extract the signal amplitude helps in lowering the noise floor as it only allows the noise at the carrier frequency to pass through while rejecting the noise at all other frequencies.

The capacitive displacement measurement mechanism is advantageous both because it offers a significant reduction in size compared to the shadow sensor (both the sensor and the readout were implemented on the same chip stack), but also because it had a displacement sensitivity that was significantly better than the optical shadow sensor. From the experimentally measured bias instability of 8.18 $\upmu$Gal for the gravimeter (see section “Allan deviation analysis”), the displacement sensitivity, $z_{\text {senst}}$, was estimated to be around 40 pm ($z_{\text {senst}} = a_{\text {bias}}/4\pi ^2f^2$). Considering the bias instability obtained from the sensor electronic noise alone, the ultimate displacement sensitivity was estimated to be just under 5 pm. These displacement sensitivity figures represent an improvement over the shadow sensor by 2 to 3 orders of magnitude. The capacitive readout methodology could be improved even further as the displacement sensitivity of this scheme ($\dfrac{\delta C}{\delta z}$, where *C* is the overlapping capacitance) is a function of the comb geometry as well as that of the gap between the two overlapping combs^39^. By increasing the comb finger density and/or bringing the proof-mass and the glass pick-up layer closer, the displacement sensitivity can be enhanced until the “pull-in” actuation limit is achieved. In the case of the proposed sensor, the minimum gap was estimated to be around 13.5 $\upmu$m. This estimation was done by re-arranging the standard pull-in voltage expression^40^ to obtain the minimum viable gap for a fixed actuation voltage: $z_{\text {min}} = \left( 27 \varepsilon _{\text {vac}} A_{\text {comb}} {\text {V}_{\text {pp}}}^2 / 8 k_{\text {oop}} \right) ^{1/3}$. Here, $k_{\text {oop}}$ is the stiffness coefficient of the first out-of-plane mode that simulations suggest to be around 80 Hz (see Table 1 for the device parameters used to evaluate the above expression). The authors opted for design parameters that provided a significant safety margin on the fabrication, assembly, and actuation tolerances. This, of course, can be optimised in the future.

To ensure the long-term stability and the noise performance of the MEMS gravimeter, it was necessary to control the temperature of the device. As discussed in Middlemiss et al.^22^, temperature fluctuations alter the Young’s Modulus, $E_{\text {Si}}$, of the silicon flexures, which in turn alters the spring constant of the MEMS device; causing spurious readings if not accounted for. For a fractional change in the Young’s modulus of Silicon, $\delta E/E_{\text {Si}}$, due to a change in temperature, the effective change in the equilibrium position can be calculated as^30,41^ $\delta z = g\left( \delta E / E_{\text {Si}}\right) / 4\pi ^2 f^2$. For Silicon, the first-order temperature coefficient of the Young’s modulus, $\beta \left( =\delta E / E_{\text {Si}} \right)$, has been stated elsewhere^42^ to be around − 60 ppm/K. Hence, for a milliKelvin change in the temperature, the equilibrium position of the proof-mass is expected to change by about 280 nm, which gives a temperature sensitivity of −58.9 $\upmu$Gal/K for the sensor. Temperature changes can also impact the thermal stability of the sensor by altering the length of the springs through the thermal expansion effect. For a monolithic construction, however, the first-order effect of thermal expansion on the spring compression is expected to be negligible^30,41^. Previous Ansys simulation studies^43^ to capture this effect on a lower frequency device (2.2 Hz) also agree with the other analytical/numerical studies and conclude that for geometric anti-springs based devices, the thermal stability is dominated by $\beta$. For the gravimeter, to limit the impact of the thermal fluctuations via $\beta$, several resistance temperature detectors (RTDs) were used to monitor the temperature of the instrument at multiple locations. The packaged MEMS sensor was fixed on to a Peltier element to control the temperature within a milliKelvin of the nominal temperature set-point.

**Table 1:** Device parameters of the MEMS gravimeter.

Parameter	Value
Frequency (*f*)	7.35 Hz
Proof mass length	12.42 mm
Proof mass height	4.82 mm
Proof mass thickness	240 $\upmu$m
Pick-up gap	30–40 $\upmu$m
Comb area ($A_{\text {comb}}$)	4.34E−05 $\hbox {m}^2$
Drive voltage ($V_{\text {pp}}$)	4 V
Drive frequency	40 kHz

More detail on the measurement set-up are provided in the “Methods” section of the manuscript.

## Results

### Earth tide measurement

One means of assessing the performance of a gravimeter is to measure the Earth tides. The Earth tides are caused by tidal forces in the Sun-Earth-Moon system^44^. The relative phase of the Sun and Moon cause the Earth to deform elastically. The effect of this deformation is a diurnal and semi-diurnal periodic variation in the distance between the crust and the centre of mass of our planet, and thus a corresponding variation in the gravitational acceleration measured at any given location on Earth. This periodic signal has an amplitude that varies between around 100 $\upmu$Gal and 300 $\upmu$Gal, depending on the monthly phase of the Moon. Maximum amplitude signals are referred to as ‘spring tides’ and minimum amplitude signals are referred to as ‘neap tides’. A measurement of the Earth tides is an ideal means of demonstrating gravimeter performance because such a measurement demonstrates both stability and sensitivity of the instrument in question. Furthermore, it is a globally recognised signal in the field of gravimetry, and it can be readily calculated using software such as T-Soft^45^. T-Soft can also be used to account for the effect of ocean loading: a local variation in the Earth tide signal caused by compression and subsequent rebound of land due to the tidal motion of large bodies of water. Ocean loading effects are more complex to calculate because they require knowledge of local topology and fluid dynamics, and can alter both the amplitude and phase of Earth tide signals by around 5$\%$.

Previous measurements have been made of the Earth tides with MEMS gravimeters. In 2016 Middlemiss et. al. published a measurement lasting 6 days^22^, with a correlation coefficient of 0.86 between the experimental data and a theoretical Earth tide calculated with T-soft. This correlation coefficient was calculated using the Pearson product-moment correlation coefficient. More recently, Tang et al.^24^ produced a device based on that of Middlemiss et al. This device was used to make a 5.5 day measurement of the Earth tides, with a correlation coefficient of 0.91 when compared to a co-located commercial superconducting gravimeter.

**Figure 2:**
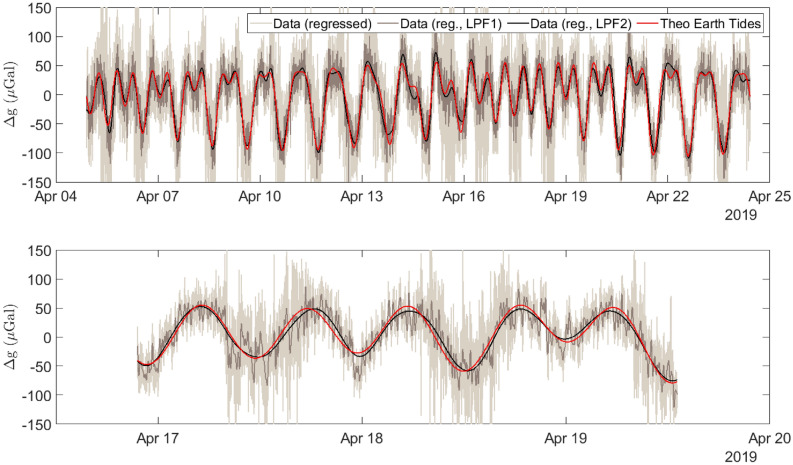
Two time series plots of the Earth tide measurement, conducted at the University of Glasgow in April 2019. In both graphs, the red series is a theoretical signal calculated using T-Soft, the gray-scale series are the experimental data recorded using the MEMS gravimeter. From lightest grey to black, these series represent increasing filtering of the data using a low-pass filter. The black series was filtered with a 20 $\upmu$Hz bandwidth low-pass filter. All of the theoretical data is presented after a regression analysis was used to remove drifts caused by temperature variations within the system. The upper graph shows the full data set, the lower graph shows a zoomed in selection of this data.

Using the MEMS gravimeter described above, a measurement of the Earth Tides was made. This data, starting on the 4th of April 2019, can be seen in Fig. 2. The measurements were taken at a sample rate of 0.18 Hz. The red series is a theoretical signal calculated using T-Soft for the measurement location (Glasgow, UK). This theoretical series includes a correction for ocean loading using the GOT00.2 model^46^. The light grey series is the unfiltered, post-regression acceleration data from the MEMS device (see “Methods” section for regression analysis details). The dark grey series shows the acceleration data after the application of a low-pass filter with a bandwidth of 1 *m*Hz. The solid black line shows the same data after a final stage of filtering, this time using a 20 $\upmu$Hz bandwidth low-pass filter. There is a correlation coefficient, *R*, of 0.975 between the theoretical data and the 2nd-stage filtered data (calculated using the Pearson product-moment correlation coefficient).

### Spectral analysis

**Figure 3:**
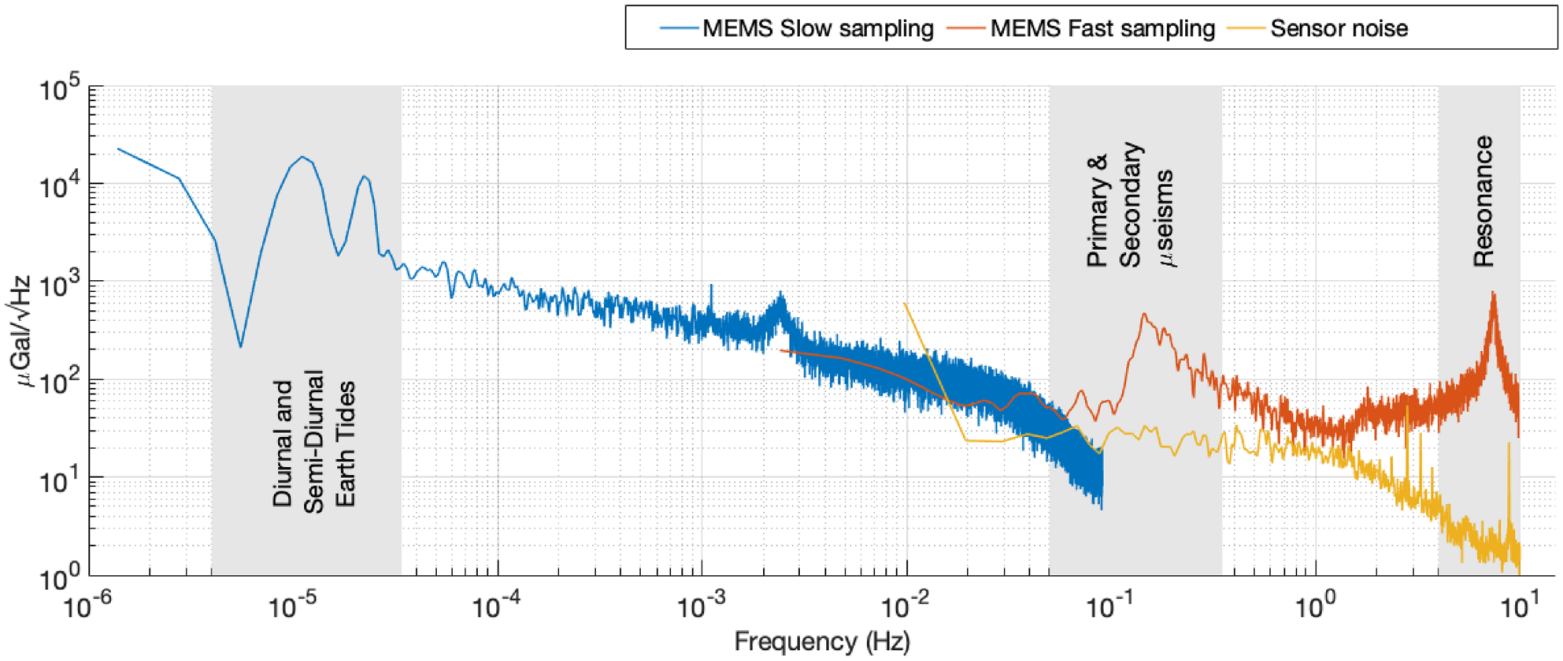
An amplitude spectral density plot of the post-regression instrument data (the blue and the orange series), and the instrument noise data (the yellow series). The blue series represents data that were recorded at a sampling rate of 0.18 Hz. The orange and the yellow series were recorded at a sampling rate of 20 Hz. Three distinct frequency bands of interest can be observed in the data—these are highlighted using the grey bands. At 10$^{-5}$ Hz, the diurnal and semi-diurnal components of the Earth tides are visible; the primary and secondary microseisms can be observed at around 0.2 Hz; and the resonant peak of the MEMS device itself is located at 7.35 Hz. The sensor noise has a flat spectral response for the measured bandwidth. *Note:* The downward roll-off at high frequencies for the blue and the yellow series is due to low-pass filtering at the demodulation stage.

The spectral analysis of the gravimeter data provides further insights into the presence of periodic signals of varying frequencies in the acceleration data. The blue series of Fig. 3 is a presentation of the drift-corrected, regressed data (the light grey series of Fig. 2) in the form of an amplitude spectral density graph. To complement the slow-sampled gravimeter data, a second series of higher-frequency gravimeter data (sampled at a rate of 20 Hz) is also included in the plot. The gravimeter data was not sampled at a higher sampling rate for the entire bandwidth to avoid unnecessary accumulation of data. This can be observed in the orange series in Fig. 3. The yellow series in Fig. 3 is the electronic noise floor of the sensor.

The blue series in Fig. 3 has a clearly identifiable double peak at $10^{-5}$ Hz. These two peaks represent the diurnal, and semi-diurnal periodicity of the Earth tides. This signal splitting occurs as the relative phase of the Sun-Earth-Moon system changes. When all are aligned in a line, the gravitational signal of the Sun and the Moon acts in phase on the Earth. When these three bodies form a right-angled triangle, however, the gravitational signal of the Sun and Moon acting on the Earth are out of phase. It is this same phase drift that causes the total amplitude to vary between spring and neap tides. Another demonstration of this Earth tide signal splitting has not before been presented in the literature (to the knowledge of the authors). Such data has not previously been presented because its observation requires the a clean time series of well over a week. In addition to the double-peak, there is a small spurious peak occurring around 2.5 *m*Hz. This signal is not geophysical in nature, and has been identified as being caused by the temperature control electronics. The roll-off seen in the slow-sampled data at higher frequencies is due to the low-pass filter operation performed by the lock-in amplifier.

In the orange series, further signals can be observed. The furthest right of these is the resonance peak of the MEMS mass-on-spring system at 7.35 Hz. This represents the maximum frequency at which the device can record meaningful data. The sensitivity to signals of higher frequencies than this resonance will rapidly decrease due to the nature of a simple harmonic oscillator. The second set of signals that can be identified in the orange series is geophysical in nature: the primary and secondary microseisms. The primary microseism occurs at frequencies between 0.04 and 0.15 Hz^47^, and is caused by interactions between ocean waves and the sea bed^48^. The secondary microseism has a larger amplitude, and occurs between 0.08 and 0.3 Hz^47^. This signal is also related to ocean activity, but is caused by interactions in shallow water between wind-driven ocean waves, and waves reflected back from the coastline. Both the primary microseisms vary in amplitude and phase, depending on ocean conditions.

As the higher-frequency band of the sensor output is dominated by the ground motion, it is difficult to estimate the true noise floor of the sensor. One way to get rid of this issue is to use a reference seismometer to effectively subtract the seisimic signals in the high-frequency band. The authors, however, did not have access to such an instrument. Instead, the sensitivity was estimated by measuring the true electronic noise floor of the full sensor set-up. This was achieved by grounding the modulation sine wave drive at the input of the MEMS capacitors. The measured sensor noise data is represented by the yellow series in the Fig. 3, and shows a flat spectral response for the measured frequency bandwidth. The roll-off observed for higher frequencies, again, is due to the low-pass filtering. The sensitivity of the gravimeter is estimated to be 18 $\upmu$Gal/$\sqrt{Hz}$ at 1 Hz. As discussed in the next section, the ultimate noise floor of the sensor is 0.91 $\upmu$Gal, which can be achieved over an integration time of 250 s.

It should also be noted that the device is also a sensitive seismometer, capable of capturing the ground motion in the vertical axis. From Fig. 2 (the light grey series), it is quite evident that the amplitude of the high-frequency components increases and then decreases across the measurement period. Since it is difficult to capture this time-localised information using standard Fourier analysis, wavelet analysis has been used to isolate the periodic variation in the high frequency noise. The details of the analysis are included in the “Methods” section.

### Stability

#### Allan deviation analysis

**Figure 4:**
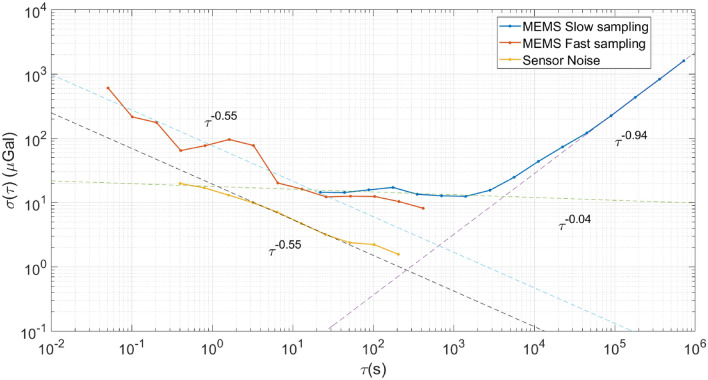
The Allan Deviation (AD) plots for the raw slow sampled MEMS data (the blue series), the raw fast sampled MEMS data (the orange series), and the sensor noise data (the yellow series). Dashed series are a result of fitting straight lines across different averaging periods for each data set. The slope of each fit is represented in the form of power-laws. *Note:* the small integration time ($\tau$) data is not shown for the blue ($\tau < 2^{2}t_{\text {slow}}$, where, $t_{\text {slow}} = 5.5$ s) and the yellow series ($\tau < 2^{3}t_{\text {fast}}$, where, $t_{\text {fast}} = 0.05$ s) as the AD values for these times are affected by the low-pass filtering stage.

To understand the stability and the noise characteristics of the gravimeter, the Allan-Deviation (AD) values ($\sigma$) were computed from the raw, uncompensated gravimeter data. The $\sigma$ values for the slow- and the fast-sampled data, and the sensor noise are plotted in Fig. 4. The fast-sampling data (the orange series in Fig. 3) has been used for evaluating the stability of the gravimeter for shorter sampling/averaging periods ($\tau$), and the slow-sampling data (the blue series in Fig. 3) for assessing the long-term stability. In the case of the slow-sampled data, the first few $\tau$ values ($\tau =$ 5.5 s, 11 s) have been excluded because the low-pass filter was shaping the frequency response. For the same reason, the first few $\tau$ values ($\tau =$ 0.05 s, 0.1 s, 0.2 s) have been excluded from the sensor noise data as well. As the fast-sampled data was logged for a sufficiently long duration, there were enough samples in the fast-sampled data to compute the $\sigma$ values for the missing $\tau$ values in the slow-sampled series.

Interpreting the noise characteristics from the AD plots is straightforward. For example, in the case of smaller averaging periods (fast sampling MEMS data, $\tau<$ 30 s), the $\sigma$ values continue to decrease, underscoring the advantage of averaging to achieve the best sensitivity of the gravimeter. Fitting a straight line through the data (dashed red line) produces a slope of $-\,0.55$, indicating the dominance of white noise^49^ for these integration times. The noise in this band is a combination of uncorrelated random walk (white noise), and correlated noise arising from the device resonance, high-frequency anthropogenic noise and the microseismic ground motion. For higher averaging periods (fast and slow sampling data, 30 s $<\tau<$ 2000 s), the band is dominated with flicker noise evident from the slope ($-\,0.04$) of the fitted straight line (dashed blue line). As the $\sigma$ values remain mostly unchanged in this band, there is no advantage of averaging the data further. The deviation values in this band are also the smallest considering all the possible averaging periods, and determine the *bias instability* of the MEMS device. The smallest $\sigma$ value, and, hence, the bias instability of 8.18 $\upmu$Gal is obtained at $\tau \sim$ 417 s when considering the fast-sampled data. Taking the slow-sampled series into account as well, the bias instability is never worse than 17.22 $\upmu$Gal (the $\sigma$ value at $\tau \sim$ 176 s). An average of all the $\sigma$ values in the band, for both the slow- and fast-sampled data, gives a *mean* bias instability figure of 13.22 (± 2.73) $\upmu$Gal between an averaging period range of 30–2000 s.

The higher averaging periods ($\tau >2000$ s) are dominated by sensor drift, mostly caused by the temperature related effects and the control instrumentation. Fitting a straight line (dashed violet line) through the last few $\sigma$ values reveal a slope of close to 1 (0.94) that is consistent with the drift rate ramp behaviour^49^. The worst drift rate of 216 $\upmu$Gals/day for the device is captured at $\tau \sim$ 1.04 days where $\sigma$ sits at 224.67 $\upmu$Gal.

The AD values obtained using the sensor electronic noise data represent the best-case scenario and can potentially be used to predict the noise characteristics of the gravimeter in the absence of any ground motion. In the case of sensor noise (the yellow series), the $\sigma$ values continue to decrease with a slope of − 0.55, obtained from fitting a straight line (the black dashed line in the figure), and reaches a minimum value of 1.6 $\upmu$Gal for a $\tau$ of $\sim$ 205 s. By extrapolating both the drift ramp from the slow sampled data (dashed blue line) and the sensor noise (dashed black line), the intersection point of the two noise regimes can be obtained. In the case of this specific sensor, that intersection point occurs at $\tau \sim$ 250 s, with an AD value of 0.91 $\upmu$Gal, that is lower than the bias instability measured just using the sensor noise data. Hence, in the absence of any ground motion, it can be concluded that the ultimate bias instability noise floor is 0.91 $\upmu$Gal.

#### Drift characteristics

**Figure 5:**
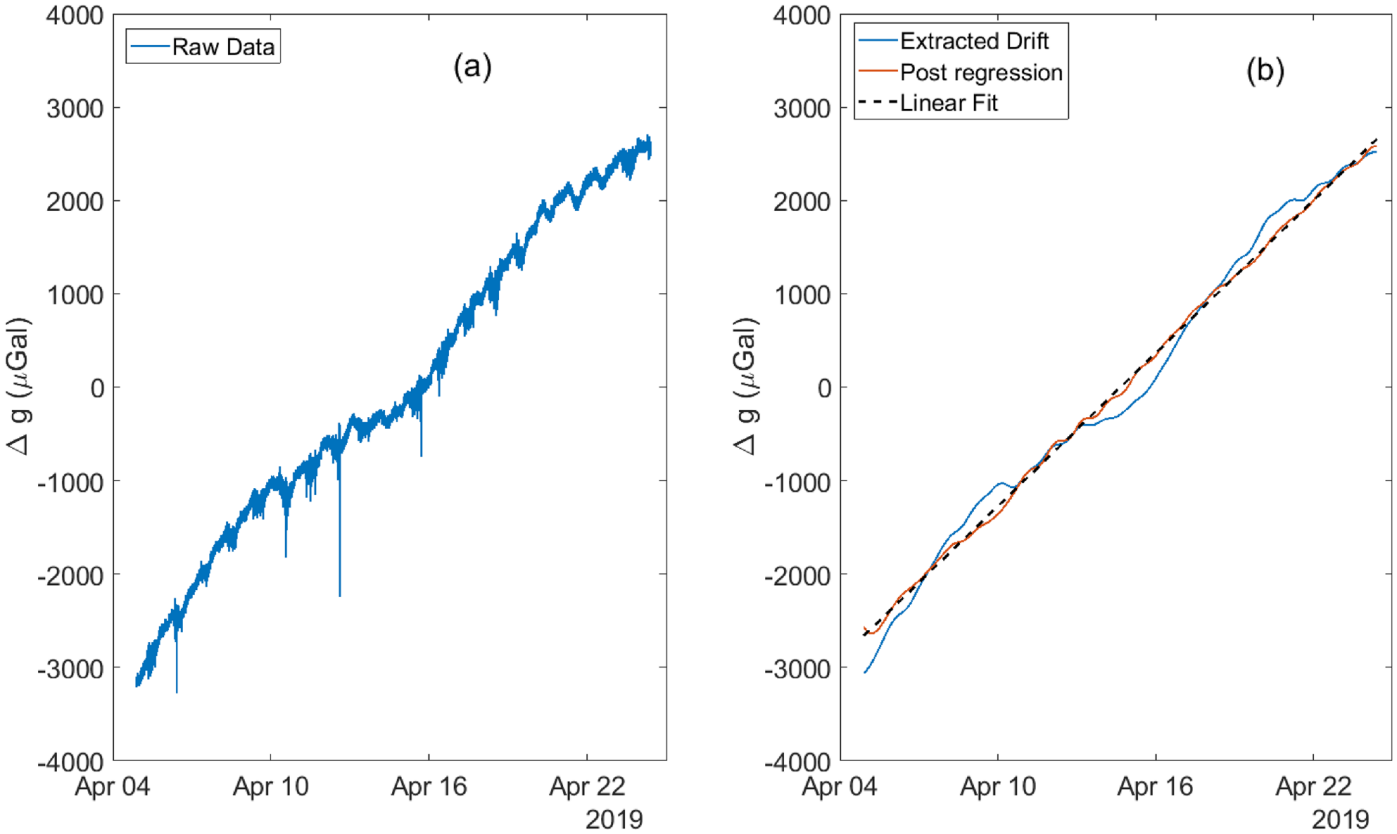
The raw data (**a**) and the drift analysis (**b**) plots for the gravimeter. The raw data is first low-pass filtered to retain only very long-term features (<2 $\upmu$Hz) in the raw data (blue series in (**b**)). The extracted drift is then processed to regress out the impact of the temperature and the control instrumentation. The post-regression data (red series) is then fitted against a straight line (dashed black series) to obtain the drift rate of the gravimeter.

As it is difficult to visualise the hidden features in the the drift of the device by simply computing the AD values, an additional time-domain analysis and polynomial curve fitting on the raw, uncompensated data to study the drift behaviour was performed. The raw sensor data is plotted in Fig. 5a and clearly shows a non-linear behaviour (the rounded ‘M’ shape) on top of a linear trend. To remove the tides from affecting the analysis, low-pass filtering, with a 2 $\upmu$Hz cut-off, was applied to the data. Setting this bandwidth was a compromise between avoiding filtering the non-linear aspects of the drift while attenuating the tide signal enough from the series. The effect of filtering can be seen in Fig. 5b, where the low-pass filtered data is represented by the blue series. In the next step, the filtered data was regressed against the temperature of the instrument and PID control output channels. Correcting for the long term temperature effects resulted in the removal of the non-linear trend leaving an almost linear drift (the red series) in the data. A straight line to the corrected data was fitted next and a linear drift of 268 $\upmu$Gal/day with an $r^2$ coefficient of 0.99 was subsequently estimated. The drift rate obtained using this analysis is slightly higher compared to the one obtained using the AD approach, and could be attributed to less than ideal signal processing (regression, fitting) of the data. It is believed that the origin of this trend is environmental (for example, temperature gradient effects that are not captured through the temperature sensors), and not geophysical.

## Conclusions

In this paper, a 19 day measurement of the Earth tides has been presented. The correlation coefficient between this measurement and a theoretical signal is 0.975, demonstrating a remarkable stability for a MEMS sensor. This sensor utilises an in-plane capacitive displacement readout, which has reduced the size of the device compared to previous work by the authors. This displacement sensor has also been used to improve the displacement sensitivity to 40 pm, leading to a bias instability value of 8.18 $\upmu$Gal without any drift correction. Processing the sensor electronic noise alone, an ultimate bias instability of <1 $\upmu$Gal was obtained. The drift rate of the device under vacuum was found to be linear and < 268 $\upmu$Gal/day. These are one of the best performance metrics in the class of MEMS gravimeters (see Table S1 in the supplementary information for a comparison with the other technologies). A network of these sensors is currently being constructed for deployment on Mount Etna as part of the NEWTON-g collaboration^20^, to create the first multi-pixel gravity imager.

## Methods

### Experimental set up

**Figure 6:**
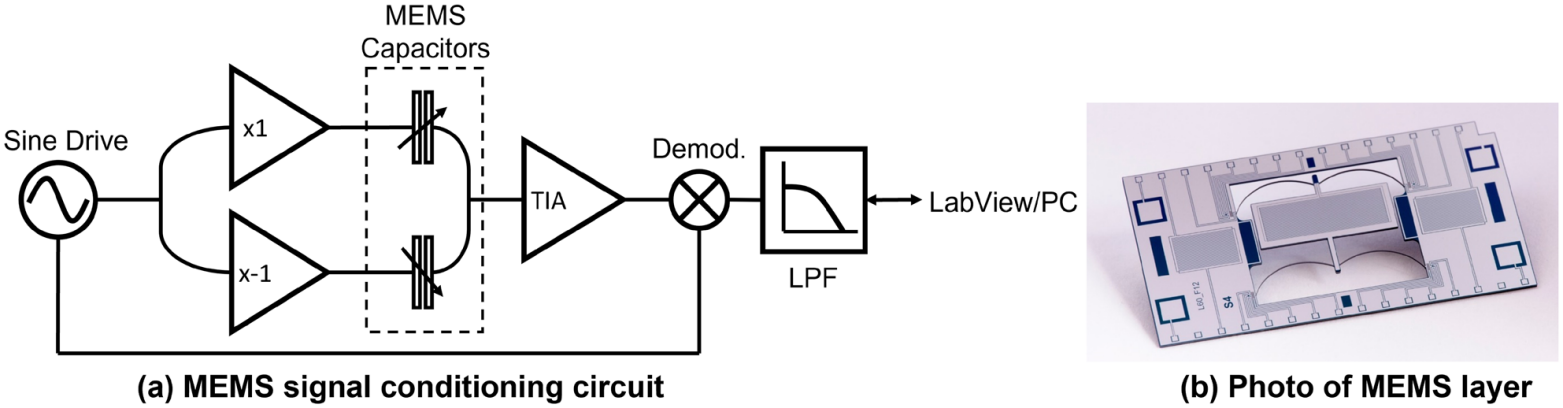
An illustration of the different components of the experimental set up: (**a**) represents the lock-in based signal conditioning of the MEMS sensor, and (**b**) is a photo of a fully-released MEMS layer (courtesy: Kelvin Nanotechnology Limited).

The MEMS device was tested within a basement laboratory at the University of Glasgow (see Fig. 6a for a schematic of the readout circuit used for taking measurements and Table 1 for details on device parameters). The three-layer MEMS device was glued inside a commercial ceramic MEMS package. The package was in turn placed inside a die-cast box, with a Peltier separating the box from the package. The die-cast box was mounted on a base plate with adjustable micrometer legs. This enabled tilt adjustment of the integrated system so that the sensitive axis of the gravimeter was aligned with the local gravitational field. The temperature of the system was controlled using three separate control loops. Two of these were monopolar Proportional Integral Derivative (PID) feedback loops, in which platinum resistors were used to measure the temperature, and two simple wire-would resistors were used to apply the temperature feedback. One pair of measurement/feedback resistors was located on the lid of the MEMS package, and one was located on the diecast box. These feedback loops were monopolar, because they can only actively change the temperature in one direction—by heating. The third loop also utilised a platinum resistor to measure the temperature, but could provide bipolar feedback: the Peltier could be used to both heat and cool. The PID control loops were used to maintain the temperature of both measurement locations at three degrees above the ambient temperature of the room. Variations in temperature were controlled to within 0.2 mK. The sensor and tilt adjustment table were enclosed within a vacuum tank. A large vacuum tank (and over-sized die-cast box) were used for this experiment so that adjustments could easily be made to the apparatus. The vacuum tank was pumped down to a pressure of around 0.1 Pa.

### Device fabrication

The MEMS device comprises three layers (as displayed in Fig. 1): a base layer, a mechanical layer, and a pick-up layer. The fabrication of three layers will be discussed in turn here.

To create the base layer a 500 $\upmu$m $\langle$100$\rangle$ silicon wafer was first coated with a layer of SPR220-7 positive photoresist. A quartz photomask was used to create the pattern for eight samples on the wafer. After development, the photoresist was used as a soft mask to etch the silicon to a depth of around 40 $\upmu$m. This etch left a depression over which the MEMS mass would be suspended.

The mechanical layer required more processing steps. Firstly, a 750 nm thermal oxide layer was grown on both sides of a 240 $\upmu$m thick, $\langle$100$\rangle$ silicon wafer. On top of the thermal oxide, a further 2 $\upmu$m of SiO$_2$ was deposited in a PECVD (plasma enhanced chemical vapour deposition) process. The top side of the wafer was then coated with two resist layers—LOR 10A and S1818. These resist layers were then patterned with using a second photolithography mask. After development, platinum was deposited in the open areas to create on-chip thermometers. Two further metalisation steps were then carried out to create NiCr heating elements, and the wiring for the capacitive sensor electrodes (using aluminium coated with a thin layer of platinum). Small areas were then chemically etched through the oxide layer, and a further metalisation step was carried out to create the connection to ground. SPR220-7 photoresist was next spun, baked, exposed, and developed to create an etch mask for the deep silicon etch. Using this resist mask, the oxide layer was selectively etched away using a CHF$_3$ plasma. The silicon was then mounted on a carrier wafer and etched using a standard Bosh etch to the full depth of the wafer, stopping on the backs-side oxide layer. To release the sample from the carrier wafer, it was soaked in methanol to dissolve the bonding compound. Finally, the back-side oxide was removed using CHF$_3$ plasma to release the sample.

The pick-up layer was made from 525 $\upmu$m thick SD2 glass. Metalisation was carried out on both sides of the sample in the same manner (again, using aluminium coated with a thin layer of platinum). The bottom side metal formed the pick-up electrodes, and the top side metal formed a ground plane. SU-8 resist was next coated on the bottom side, it was then baked, exposed, and developed to create 30 $\upmu$m tall spacers. These spacers separated the pick-up layer from the mechanical layer then the three layers were bonded together using silver epoxy. A process flow diagram illustrating the various fabrication steps is presented in Fig. S1 of the supplementary information to aid the readers.

### Data analysis

#### Regression analysis

Standard multiple linear regression analysis to remove the instrument noise from the MEMS data. The long time-period data was collected/split with samples roughly of 2 days duration. The regression analysis was carried out on each segment followed by stitching to generate a continuous sample of roughly 19 days. The first couple of days worth of data was not used in the analysis as the state of the experimental set up was found to be transitionary, and reaching a quasi-equilibrium state, during the evacuation cycle. The observed MEMS data was regressed against three temperature channels, two temperature control servo outputs, tilt, theoretical tides and a 3rd order polynomial drift model. The regression routine was implemented on Matlab using the software’s in-build functions. The regression analysis also provides with a calibration factor for the device which comes out to be around 7.83 (± 0.54) $\upmu$Gal$/\mu$V.

#### Tidal correlation analysis

To assess the coherency that the measured gravity has with the synthetic tide data, a cross-correlation analysis was carried out. The time-lag at which the two time-series are best correlated can also be ascertained. A Pearson product-moment correlation coefficient was calculated between the theoretical Earth tide data, and the 20 $\upmu$Hz filtered data from Fig. 2. This produced a correlation coefficient of 0.975 and a time lag of 15.4 min. This prompted the question of whether there was a possible issue with the synthetic Earth tide model chosen in T-Soft (which uses the OSU TPXO9 atlas^50^). To address this, cross-correlation results were assessed over 24-h consecutive windows throughout the full record. The results of this are included in the upper plot of Fig. S2 of the supplementary information. It can be observed that most of the values remain consistently high. Days 15 and 16, however, show a drop in the coefficient to approximately 0.90. This is synchronous with an apparent lag in the data (as can be observed in the lower plot of Fig. S2 in the supplementary information). For days 15 and 16 this is 17.4 and 14.0 min respectively. Such a lag cannot be explained by an incorrect application of ocean loading: if the source were ocean-loading related, one would expect the lag to be observed uniformly over the entire series. The only possible exception to this would be a weather-related anomaly (a storm surge for example), but no such weather event took place, nor would one expect this to cause a lag of this magnitude. It can be concluded, therefore, that this dip in correlation coefficient and coherency is due to a temperature related anomaly.

#### Wavelet analysis

**Figure 7:**
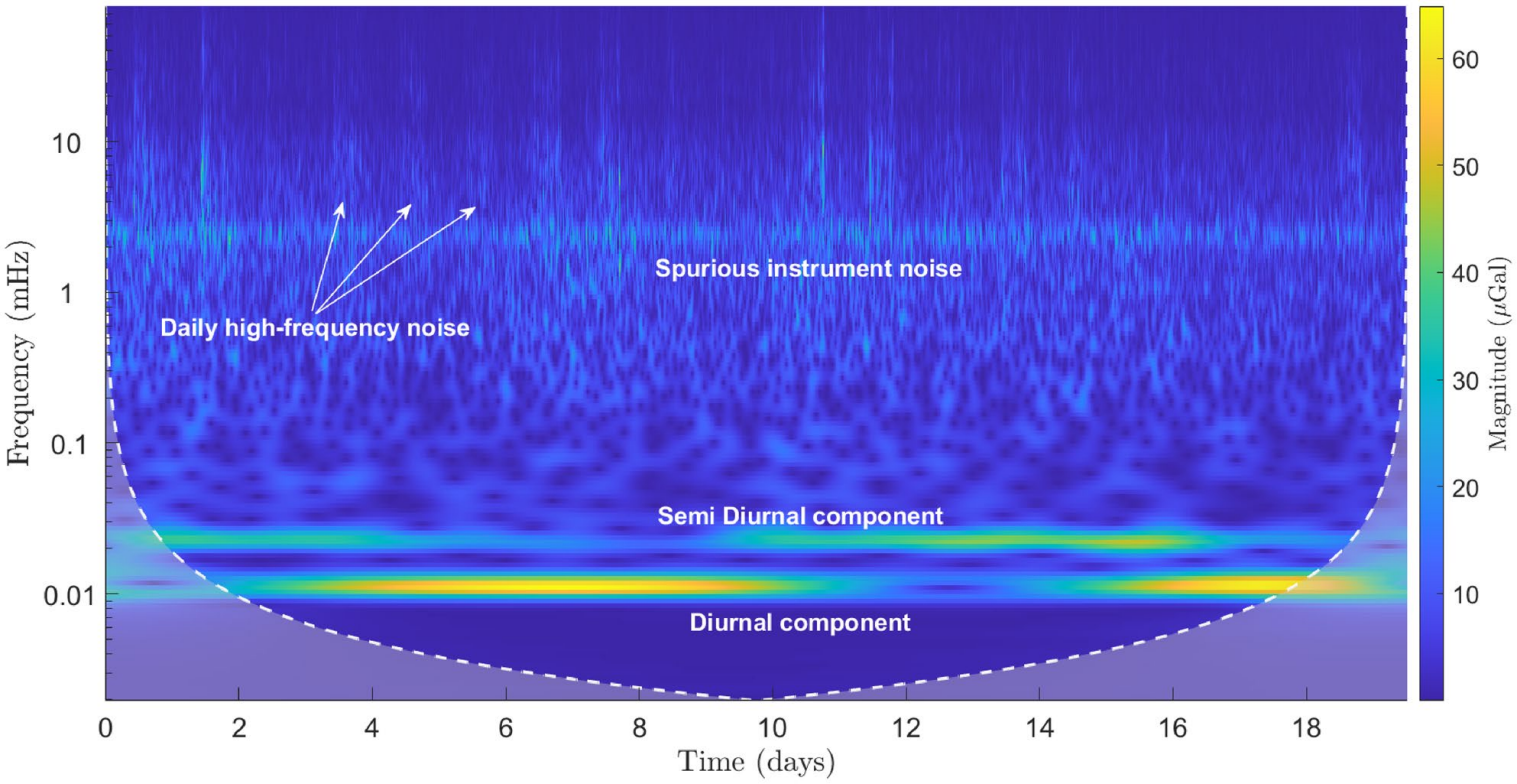
A scalogram graph of the MEMS data generated using continuous wavelet analysis on Matlab. The double Earth tide peaks are visible at the lower frequencies while the higher frequency band consists of a continuous spurious signal arising from the temperature control electronics and intermittent high frequency noise.

As noted earlier, standard Fourier analysis is not ideal for isolating non-stationary signals embedded in a time series. An alternative is to perform Short-term Fourier Transform (STFT) to obtain time localised information on signals of interest. However, STFT suffers from the limits imposed by the uncertainty principle which dictates that both temporal and frequency information for a signal cannot be obtained with a high degree of resolution at the same time. A better solution in such a case is to carry out a Multiresolution Analysis (MRA) using wavelets. The MRA is particularly useful when trying to resolve the higher frequency signals that occur over short time periods or low frequency signals that occur over long periods. As the data is contaminated with large amplitude spikes of significant energies (for example, spikes on 6th, 10th, and 12th April in Fig. 5a), to resolve smaller amplitude signals with a good contrast, the data was first de-spiked using a median filter. The median filter is preferred over a standard averaging filter (low-pass filter) as it only acts on spikes, leaving the other lower amplitude signals unaffected. A wavelet magnitude scalogram using continuous wavelet transform was generated next (in Fig. 7) using the default Morse wavelet on Matlab. In the scalogram, the semi diurnal and diurnal components due to the Earth tides are clearly visible. The phase variation between the two signals is also evident in the figure. The instrument noise that is endemic to the measurement, and which occurs at 2.5 *m*Hz, is also clearly visible. Interestingly, a band of high frequency signals (> 2.5 *m*Hz) localised only on particular days of the measurement record can also be seen. These high frequency signals, as expected, were not resolved in the amplitude spectral density plot in Fig. 3.

To further analyse the daily high-frequency noise in the data, the regressed data (without the spike removal filter applied to it) was decomposed down to a scale of 8 with a sym4 wavelet through a maximal overlap discrete wavelet transform routine on Matlab. The first two detail coefficients (D1 and D2) along with their corresponding zoomed in versions have been plotted in Fig. S3 of the supplementary information. The light orange rectangles in (a) and (c) depict the workweeks (Mon–Fri) in the month of April, 2019. The high frequency noise are observed mostly during the workweek and are roughly localised within 10–12 h window (starting roughly after 7 am and ceasing by 6 pm) (see (c) and (d) in the figure). The source of the noise seems anthropogenic due to their daily occurrence during the working days and limited strength during the weekends. Incidentally, during the measurement period, heavy construction work was being carried out at two different sites that are < 150 m away from where the device was located. The authors believe that the high-frequency noise in the data was due to excavation work being carried out during the daytime at the two sites (The Advanced Research Centre and the Jame McCune Smith Learning Hub within the University of Glasgow’s Gillmorehill campus).

## Supplementary Information

Supplementary Information.

## Data Availability

The research data relevant to this letter are stored on the University of Glasgow’s Enlighten Repository.
