# Supplementary material for: A 19 day earth tide measurement with a MEMS gravimeter
Source: Sci Rep. 2022 Jul 29;12:13091. doi: 10.1038/s41598-022-16881-1 (PMC9338064; doi:10.1038/s41598-022-16881-1)
Supplement: Supplementary file 1 — Supplementary Information. [file 41598_2022_16881_MOESM1_ESM.pdf]

A Nineteen Day Earth Tide Measurement with a MEMS Gravimeter
Supplementary Information

Abhinav Prasad*[1], Richard P. Middlemiss*[1], Andreas Noack[1], Kristian Anastasiou[1], Steven G. Bramsiepe[1], Karl Toland[1], Phoebe R. Utting[1], Douglas J. Paul[2], and Giles D. Hammond[1]

[1]School of Physics and Astronomy, University of Glasgow, Kelvin Building, University Avenue, Glasgow, G12 8QQ, U.K.
[2]James Watt School of Engineering, University of Glasgow, Rankine Building, Oakfield Avenue, Glasgow, G12 8LT, U.K.

June 27, 2022

S1. Fabrication Process Flow Diagram

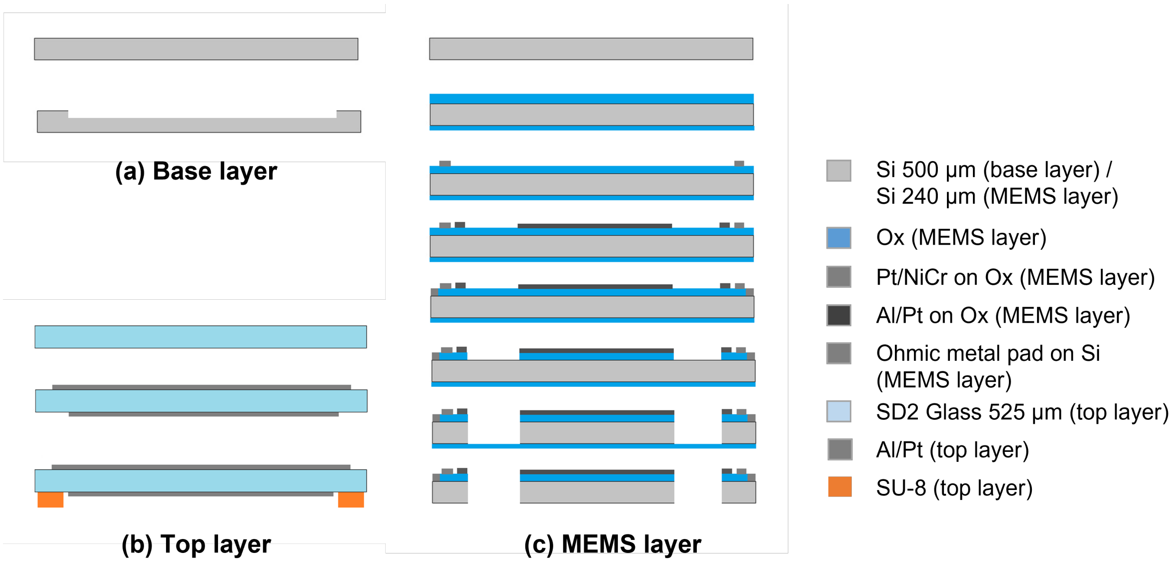

Figure S1: The process flow for the fabrication of the base layer (a), the top layer (b), and the MEMS layer (c). Each schematic sequentially defines (from top to bottom) the steps taken in their fabrication, as described in Sec 5.2.

S2. Tidal Correlation Analysis Plots

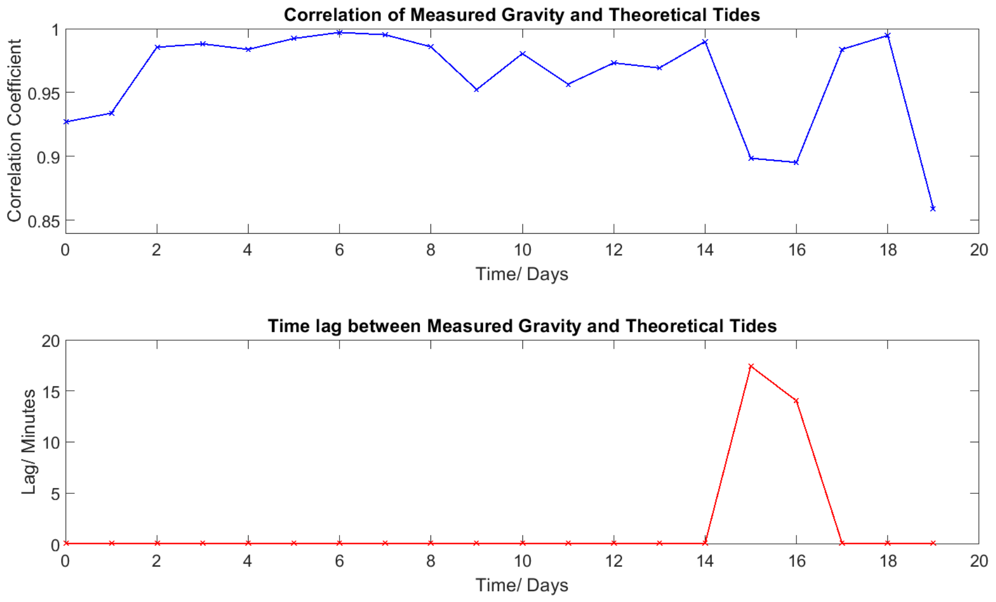

Figure S2: In the top figure, the correlation coefficients between the MEMS data and the synthetic tide signal are plotted for every measurement day. In the bottom figure, the lag observed between the two signals is plotted against time.

S3. Wavelet Analysis Plots

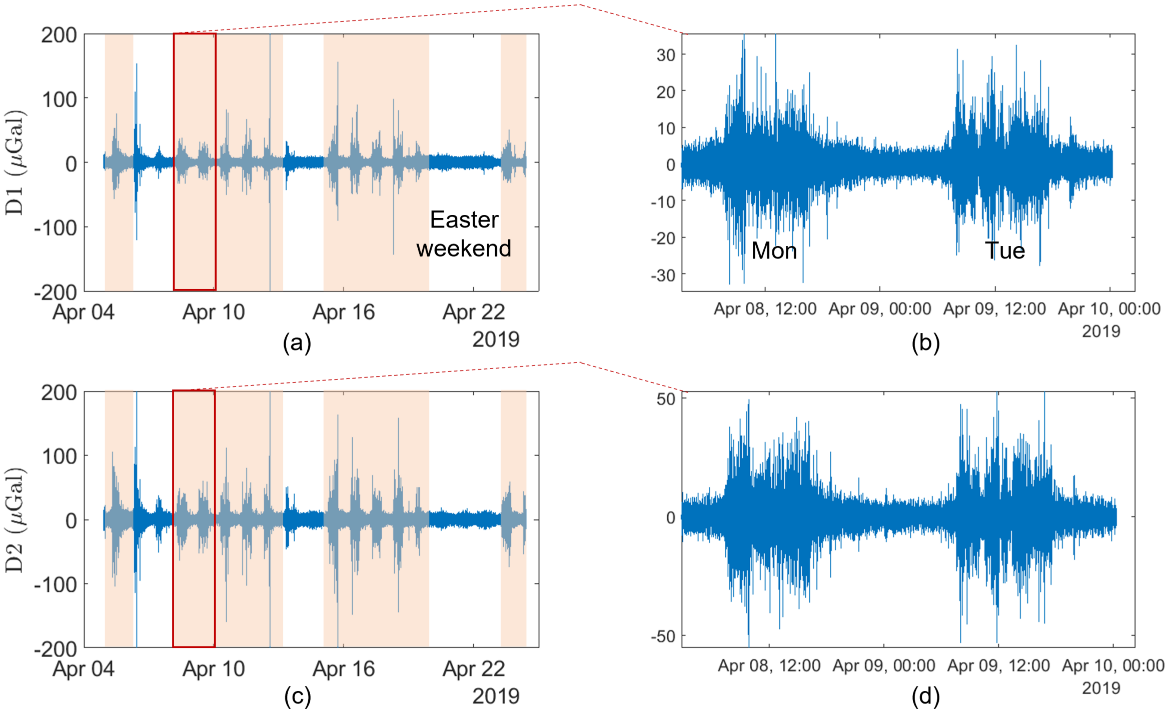

Figure S3: Each row represents the detail coefficients extracted after carrying out Multiresolution Analyses of the regressed data using Discrete Wavelet Analysis. The top row represents the highest scale level (D1) while the second row represents the next scale level (D2). In each row, te left figure contains the coefficient for the entire duration, while the right figure represents the zoomed in data over a small section of time series. The shaded regions in (a) and (c) indicate workweeks (Mon-Fri) while the un-shaded regions indicate weekends/holidays. The high frequency noise seen in the graphs were found to coincide with the usual work days, typically starting in the morning (7-8 am) and ending close to 6 pm.

S4. Other Gravimeter Metrics

The quality factor (Q) of the device when operated in vacuum was observed to be around 25. This value was obtained by fitting a Lorentzian function to the resonance peak data that was measured using fast-sampling. The dynamic range of the sensor, while not measured experimentally, could be estimated by taking a few assumptions. The first assumption is that the sensor was operating at the optimal loading point of its frequency-loading curve. This ensures a Hook's law (or, linear) spring behaviour of the flexures for a reasonable proof mass displacement (typically hundreds of microns)[2]. The second assumption is that the proof mass displacement remains within a range where the capacitive gradient ($\delta C/\delta z$) can be considered more or less the same. As the capacitive gradient follows a cosine behaviour (maximum magnitude at the geometric null points of the overlapping combs where the differential MEMS capacitors cancel each other out, and minimum magnitude where one of the differential capacitors maximise), it is assumed that 1/8th of the comb pitch above and 1/8th of the comb pitch below the geometric null point gives a good approximation of the aforemen-

tioned displacement regime. This means, if the proof-mass is centered at the null point, it can displace $\pm$ 18.75 μm without invoking the readout non-linearity. For this specific sensor, this is estimated to be around $\pm$4 Gal under a 1g DC loading.

References

[1] *CG-6 Autograv Gravity Meter*. https://scintrexltd.com/product/cg-6-autograv-gravity-meter/. Accessed: 2022-06-08.

[2] RP Middlemiss et al. “Measurement of the Earth tides with a MEMS gravimeter”. In: *Nature* 531.7596 (2016), pp. 614–617.

[3] Arif Mustafazade et al. “A vibrating beam MEMS accelerometer for gravity and seismic measurements”. In: *Scientific reports* 10.1 (2020), pp. 1–8.

[4] William T Pike et al. “A self-levelling nano-g silicon seismometer”. In: *SENSORS, 2014 IEEE*. IEEE. 2014, pp. 1599–1602.

[5] Shihao Tang et al. “A high-sensitivity MEMS gravimeter with a large dynamic range”. In: *Microsystems & nanoengineering* 5.1 (2019), pp. 1–11.

S5. Performance comparison with the other technologies

**Device**	**Technology**	**Bias instability / Sensitivity**	**Drift rate (/day)**	**Tides matching**	**Use**
Scintrex CG-6[1]	Fused Quartz	<5 μGal std. dev.	<200 μGal drift uncorrected	-	Gravimetry
Tang et al[5]	MEMS; Optical; 3.1 Hz resonant frequency	13.5 μGal ($\tau = 20$ s)	2.4 mGal	5.5 days (0.91 corr.)	Gravimetry
Cambridge[3]	MEMS; Frequency tracking	9 μGal ($\tau = 1000$ s) drift corrected; >20 μGal ($\tau = 70$ s) drift uncorrected	16 mGal	3.5 days (0.82 corr.)	Gravimetry
Imperial[4]	MEMS; Capacitive; 11 Hz resonant frequency	2 μGal/$\sqrt{\text{Hz}}$ at 10 Hz sensor self-noise	-	-	Seismometry
Glasgow[2]	MEMS; Optical; 2.3 Hz resonant frequency	40 μGal/$\sqrt{\text{Hz}}$ at 1 Hz	150 μGal	5 days (0.86 corr.)	Gravimetry
This work	MEMS; Capacitive; 7.35 Hz resonant frequency	8.18 μGal ($\tau = 417$ s) drift uncorrected; <1 μGal ($\tau = 250$ s) sensor electronic noise	<268 μGal	19 days (0.975 corr.)	Gravimetry

Table S1: A table comparing the proposed MEMS gravimeter with other relative gravimeter technologies. Note: the fields which are left blank either mean that particular information is not publicly available or the information is not available in the cited source.
